# Dihydroartemisinin attenuates hypoxic-ischemic brain damage in neonatal rats by inhibiting oxidative stress

**DOI:** 10.1186/s13041-022-00921-y

**Published:** 2022-04-28

**Authors:** Qian Xiong, Xiaohuan Li, Lei Xia, Zhengyu Yao, Xiuyu Shi, Zhifang Dong

**Affiliations:** Pediatric Research Institute, Ministry of Education Key Laboratory of Child Development and Disorders, National Clinical Research Center for Child Health and Disorders, China International Science and Technology Cooperation Base of Child Development and Critical Disorders, Chongqing Key Laboratory of Translational Medical Research in Cognitive Development and Learning and Memory Disorders, Children’s Hospital of Chongqing Medical University, Chongqing, 400014 China

**Keywords:** Hypoxic-ischemic brain damage, Dihydroartemisinin, Oxidative stress

## Abstract

Neonatal hypoxic-ischemic encephalopathy (HIE) induced by perinatal asphyxia is a major cause of neurological disability among infants. Dihydroartemisinin (DHA), derived from artemisinin, well known as an anti-malarial medicine, was proved to be able to inhibit oxidative stress and inflammation. However, whether those functions of DHA play roles in hypoxic-ischemic brain damage (HIBD), an animal model of HIE in patient which also been observed to have oxidative stress and inflammation, is unknown. In this study, we demonstrated that the DHA treatment on newborn rats significantly relieved the neuron loss and motor and cognitive impairment caused by HIBD. One of the underlying mechanisms is that DHA enhanced the anti-oxidant capacity of HIBD rats by up-regulating the total antioxidant capacity (T-AOC), gluathione reductase (GR) and catalase (CAT) while down regulating the pro-oxidative substances including hydrogen peroxide (H_2_O_2_), total nitric oxide synthase (T-NOS) and inducible nitric oxide synthase (iNOS). Thus, our study illustrated that DHA could alleviate the damage of brains and improve the cognitive and motor function of HIBD rats by inhibiting oxidative stress, provided an opportunity to interrogate potential therapeutics for affected HIE patients.

## Introduction

Perinatal asphyxia induced series clinical manifestations of central nervous system usually leading to HIE. It is the second most common cause of neonatal death after complications of premature delivery and accounts for about 25% of neonatal mortality [1, 2]. Previous reports showed that the prevalence rate of HIE which cause long-term cognitive impairment of children was 1.5‰ [3]. Patients with severe HIE are often suffer from sequelae as cerebral palsy, epilepsy and learning difficulties [4]. However, mild hypothermia therapy as one of the most common used therapies for HIE is not efficient for all patients [5]. Developing novel treatment to improve therapeutic becomes urgent.

HIBD is reported to be related with inflammation and activated glial cells, which generate pro-inflammatory factors such as cytokines, chemokines, nitric oxide synthase (NOS) and reactive oxygen species (ROS), affecting the development of brain and causing short-term or long-term harmful effects [6, 7]. Moreover, oxidative stress is also reported to be involved in promoting the progression of HIBD [8, 9]. When hypoxia and ischemia (HI) occurs, with the immature antioxidant defense system and deficient energy, active oxygen accumulates in the body of newborns and exceeds the normal level. This can modify or destroy cell macromolecules directly, lead to cascade inflammatory reactions and protease secretion, change cell structure or function and damage cerebral tissues through various ways [10–12]. Continuous monitoring of the central nervous system of HIE patients showed that GSH, a biomarker of oxidative stress which plays a neuroprotective role by eliminating reactive oxygen species in tissues [13, 14], significantly decreased in the damaged area. All above suggest that suppressing the oxidative stress in HIE could be a promising treatment for it.

Interestingly, we noticed that Artemisinin as a well-known antimalarial drug has been reported to play anti-inflammatory and anti-oxidative roles in many diseases [15–19]. Some studies have shown that artemisinin can activate Adenosine 5 ‘-monophosphate-activated protein kinase (AMPK) in the presence of H_2_O_2_, protect SY5Y cells and hippocampal neurons, promote ROS to normal level and reduce the occurrence of apoptosis [20]. In the model of hepatic encephalopathy, artemisinin inhibits the oxidative damage of neurons induced by ammonia and improves the glutamate signal in astrocytes, restoring the damaged spatial learning ability [21]. In mice with Alzheimer’s Disease, artemisinin increases the expression of superoxide dismutase (SOD) and induces phosphorylation of AMPK/GSK3β pathway, then increases the level of antioxidant protein heme oxygenase-1 (HO-1) after activating nuclear factor erythroid-2-related factor 2 (Nrf2), which inhibits apoptosis of cortical neurons and activation of glial cells to alleviate learning and memory impairment [22]. DHA, a derivative of artemisinin, has similar physical and chemical properties and functions. It can combine with plasma proteins in vivo and penetrate the blood–brain barrier [23]. A recent study showed that DHA can inhibit neuronal apoptosis and reduce the loss of neurons in APP/PS1 mice by promoting the expression of brain-derived neurotrophic factor (BDNF) and neuroplasticity-related proteins [24]. In acute kidney injury model, DHA protects the kidney by inhibiting nuclear factor kappa B (NF-κB)-mediated inflammation and oxidative stress [25]. Besides, it reported that DHA significantly increased the level of SOD and GSH in bleomycin-induced pulmonary fibrosis of rats through Nrf2/HO-1 signaling pathway [26]. However, the effects of DHA on HIBD rats are still unknown.

DHA's potential anti-oxidative stress characteristics inspired us to ask whether it could alleviate brain injury and improve the prognosis of patients with HIE. In this study, we demonstrated the protective effect of DHA on the neonatal rats after HI by behavioral tests, immunofluorescence staining and enzyme-linked immunosorbent assay (ELISA).

## Materials and methods

### HIBD modeling

The surgery for HIBD modeling were proceeded at postnatal day 7 (P7) of Sprague–Dawley (SD) rats. Specifically, the left common carotid arteries were ligated. Two hours after the surgery, rats were placed in an enclosed container with 8% oxygen and 92% N_2_ maintaining for 2.5 h for hypoxia [27]. The sham group were only proceeded left common carotid arteries isolation without hypoxia treatment. Pups were placed back to their home cage with dams after processing.

### DHA treatment

**Fig. 1:**
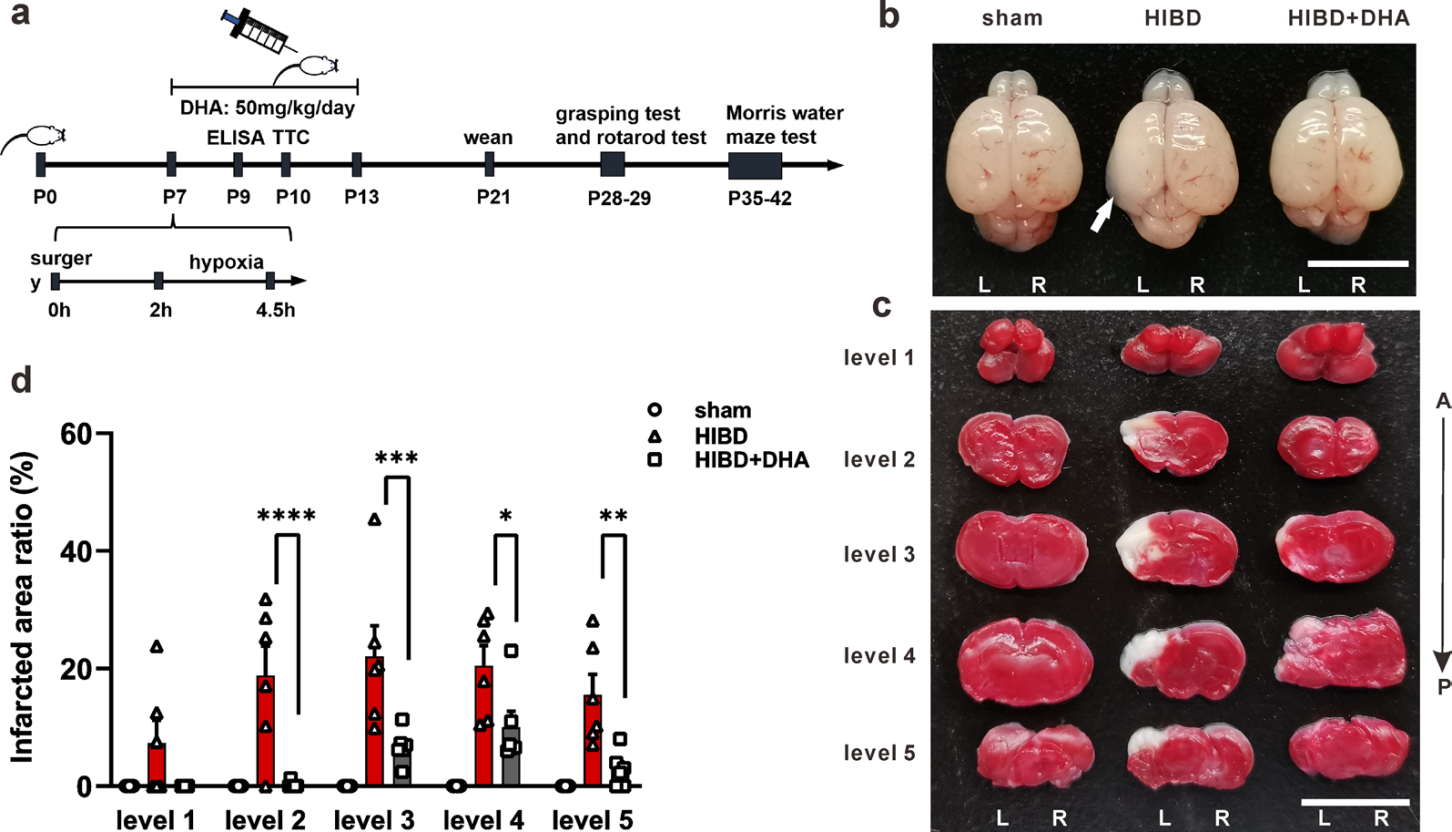
DHA treatment reduces the HI induced infarction. **a** A schematic of experiments time line. **b** Representative dorsal views of rat brains (P10, 72 h after HI/sham surgery). White arrow indicates the liquefactive necrosis area. **c** Representative TTC staining coronal sections (2 mm) of brains from panel **b**. Sections are labeled as five different levels (level 1–level 5) along the anterior (“A”) to posterior (“P”) axis. **d** Quantification of the cerebral infarct area in brain sections (infarcted area% = white (stainless) area/total area of slice). L: left, R: right. Scale bars = 1 cm

DHA dissolved in DMSO (diluted to 9.5 mmol/L with saline as working solution) was administered intraperitoneally (50 mg/kg) [26, 28, 29] once a day for 7 days to the experimental groups of rats started right after HI modeling. The same dose of DMSO + saline was intraperitoneally injected to the relative groups as a control (see Fig. 1a).

### TTC staining

Brains of P10 rats (3 days after HI modeling) were harvested for TTC (2,3,5-triphenyltetrazolium chloride) staining. Five coronal sections were taken for each brain after frozen at − 20℃ for 20 min. The incisions were at the middle of forebrain and optic chiasma, optic chiasma, funnel stalk, and the midpoint of funnel stalk and caudal pole of posterior lobe. Slices were placed in 2% of TTC solution at 37℃ for 20 min with flipping for every 5 min followed by PBS washing and photographing [30].

### Grasping test

Myodynamia of rat forelimbs were detected by a grip tester with 5 times of duplicated tests for each limb. Left and right forelimbs of P28 rats (21 days after HI modeling) were placed on the grip detector respectively.

### Rotarod performance test

Locomotor condition of rats was assessed on an accelerating rotarod [31] after griping tests. Specifically, rats (P29) were put on the rotarod twice (rotary speed is 0 rpm and 20 rpm, respectively) one day before formal tests as the pre-training session. Two trials (cut-off time: 3 min) with 20 min of interval were given to the rats at the following day, in which speed accelerated from 5 to 50 rpm and the latency to fall was recorded.

### Water maze test

To assess the spatial learning and memory of rats, Morris water maze was used as reported with modifications [32]. Briefly, the water maze consists of a black circular pool (180 cm diameter, 60 cm height) filled with water with nontoxic black dye (22 ± 1 ℃) to the level of 40 cm. The pool was divided into four equal quadrants, designated as Northeast (NE), Southeast (SE), Southwest (SW) and Northwest (NW), and a platform (12 cm diameter) was placed 1 cm below the water surface at SE quadrant. Rats(P35) were put into the pool for 60 s from the midpoint of the NW at first day for environmental adaptation, trained to navigate and find the submerged platform for four times/day during the next 5 days (day1 to day5). Rats were guided to the platform and allowed to stay on it for 10 s if they failed to find the platform during the training (cut-off time: 60 s). Then, the platform was removed after 5-day training and probe test was performed. The number of times that rats crossed the platform position, the time they spent to arrive the previous platform position and the total time they stayed in the target quadrant were recorded. The whole process was recorded by Any-maze tracking system.

### Immunofluorescence

Immunofluorescence (IF) were performed as described with modifications [33]. Adult rats (P42) were cardio-perfused with phosphate buffered saline (PBS), the left hemispheres of rats were then collected and fixed in 4% paraformaldehyde (PFA) for 5 days (4 ℃). After washed with PBS, the brains were transferred to 30% sucrose solution until they all sank to the bottom of the container. Cryo-sectioning of 40 μm along the coronal plane were proceeded and sections were blocked with blocking buffer (Beyotime) for 1 h (room temperature, RT) followed with NeuN antibody incubation (1:200, abcam) at 4 ℃ overnight (every sixth slice with the same reference position was stained). Secondary antibody, Alex 488 (1:200, abcam), was applied next day for 2 h (RT). Sections were then mounted with DAPI included medium (Beyotime). IF images were captured by a microscope (Leica Stellaris 5 WLL) at 20 × magnification.

### Oxidative stress relative tests

Different groups of brains (hippocampus and cortex) were separately collected at 48 h after HI modeling, followed by homogenization with tissue/saline rate of 1: 9 under the condition of ice-water bath. The lysates were then centrifuged to collect the supernatant. ELISA for oxidative stress related factors were performed with the previous supernatant respectively by ELISA kits (Nanjing Jiancheng) as their instructions. The factors we tested were as follows: T-AOC, GR, CAT, H_2_O_2_, TNOS and iNOS.

### Image analysis

The infarct area measurement by TTC staining and the counting of NeuN^+^ cells were performed with Image J.

### Statistical analysis

All statistical analyses were conducted with SPSS 25 software or Prism 9.2.0. All graphs were plotted as mean ± the standard error of the mean (SEM) with Prism 9.2.0 software. *p < 0.05, **p < 0.01, ***p < 0.001, ****p < 0.0001.

## Results

### DHA treatment alleviates cerebral infarction in HIBD rats

Given the liquefactive necrosis occurs in brain when hypoxia and ischemia happen, we first used TTC staining to evaluate the HIBD modeling. As expected, the brains after HI showed obvious liquefactive focus (Fig. 1b) and significant portion of stainless area representing the location of infarction (Fig. 1c, d). Notably, the infarcted area ratio (white infarcted area/brain slice area) was significantly reduced if rats were treated with DHA after the HI surgery (Fig. 1c, d) (level 1: sham = 0, HIBD = 7.30 ± 3.91%, HIBD + DHA = 0, p_HIBD vs. HIBD+DHA_ > 0.05; level 2: sham = 0, HIBD = 18.84 ± 4.96%, HIBD + DHA = 0.22 ± 0.22%, ****p_HIBD vs. HIBD+DHA_ < 0.0001; level 3: sham = 0, HIBD = 22.08 ± 5.18%, HIBD + DHA = 6.71 ± 1.16%, ***p_HIBD vs. HIBD+DHA_ = 0.0004; level 4: sham = 0, HIBD = 20.46 ± 3.46%, HIBD + DHA = 10.02 ± 2.71%, *p_HIBD vs. HIBD+DHA_ = 0.0208; level 5: sham = 0, HIBD = 15.51 ± 3.50%, HIBD + DHA = 2.89 ± 1.22%, **p_HIBD vs. HIBD+DHA_ = 0.0041; n = 5, 6, 6 for sham, HIBD, and HIBD + DHA group, respectively; Two-way ANOVA, Tukey’s multiple comparisons test). This phenotype suggest that HI-induced necrosis could be largely prevented by DHA treatment (Fig. 1b, c).

### DHA treatment prevents the HIBD-induced neuron loss

**Fig. 2:**
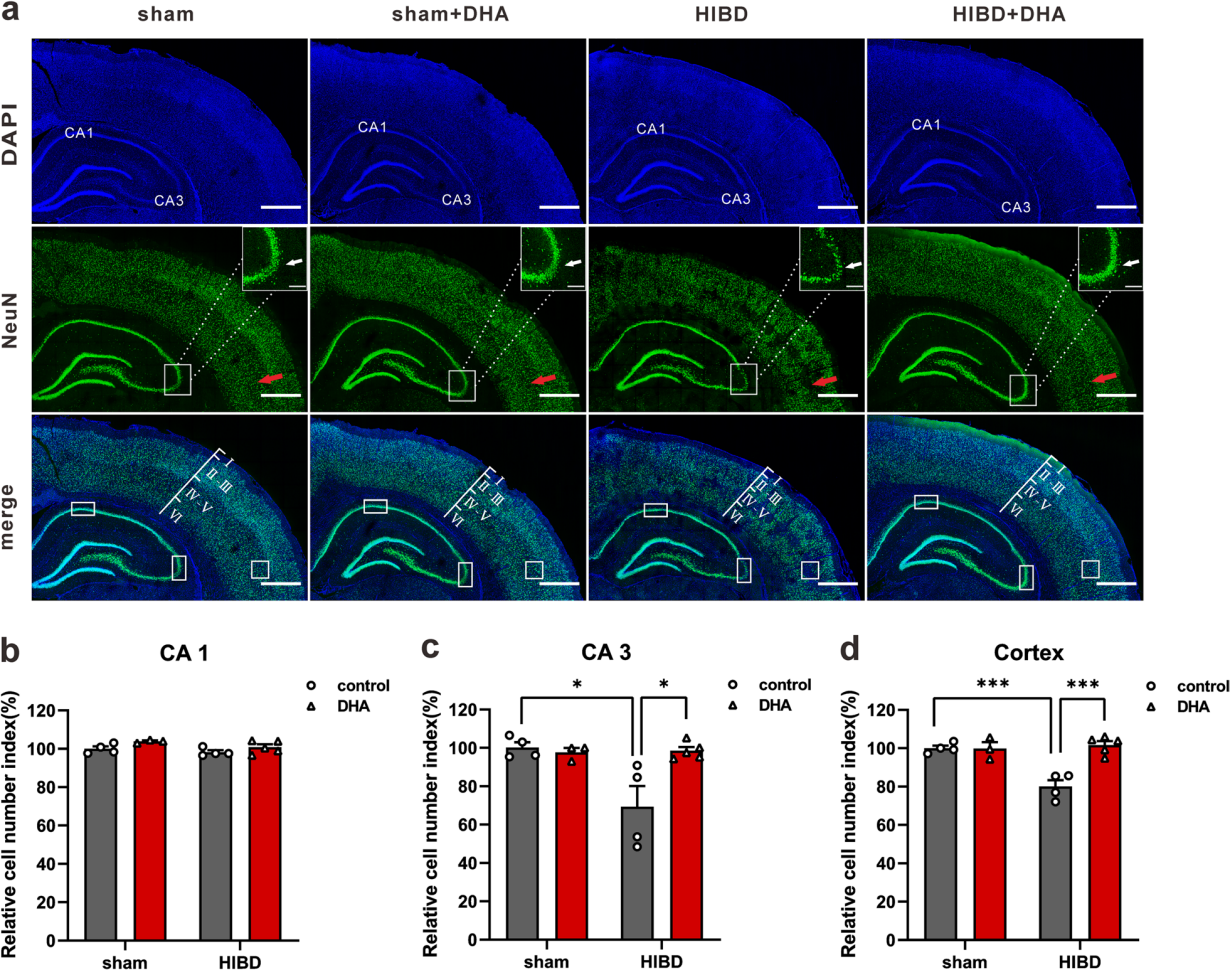
DHA treatment reduces the HI induced neuronal loss. **a** NeuN immunofluorescence in adult (P42) cortex and hippocampus (coronal). Green: NeuN, blue: DAPI. Red arrows indicate the NeuN^+^ cell loss in cortex (layer I–VI are labeled). Images in the boxes at the right top corner of NeuN chenel: magnified images of each boxed area. White arrows in the magnified images indicate the NeuN^+^ cell loss in CA3 areas of hippocampus. Rectangles in the images of merged chenel indicate the ROI for quantification (cortex: 602.5 μm * 605 μm * 6 per rat; CA1: 500 μm * 185 μm * 6 per rat; CA3: 310 μm * 720 μm * 6 per rat). Scale bars = 1 mm (200 μm for the magnified images). **b–d** Quantitation of NeuN^+^ cell numbers in the rectangles of panel **a**. Relative cell number index = NeuN^+^ cell numbers/average NeuN^+^ cell numbers of sham group%

We next asked whether the number of neurons might be smaller, since HI is known to induce infarction, and children with HIE are known to have mental retardation [34]. Immunofluorescence staining of NeuN, a biomarker for mature neurons, with frozen sections of adult rat brain showed that the number of neurons in hippocampal CA3 area in HIBD group were significantly decreased compared to the ones in the sham group. Notably, this neuron number decrease could be dramatically rescued by DHA treatment (Fig. 2a, c). Relative cell number index (RCNI) = cell number in region of interest (ROI)/average cell number of ROI in sham group. (For CA3 area, RCNI^sham^ = 100 ± 1.98, RCNI^sham+DHA^ = 97.65 ± 1.47, RCNI^HIBD^ = 69.38 ± 4.16, RCNI^HIBD+DHA^ = 98.44 ± 1.24; n = 4, 3, 4, 5 for sham, sham + DHA, HIBD, and HIBD + DHA group, respectively; *p_sham vs. HIBD_ = 0.0115, *p_sham+DHA vs. HIBD_ = 0.0320, *p_HIBD vs. HIBD+DHA_ = 0.0120; Two-way ANOVA, Tukey’s multiple comparisons test). Interestingly, the cell number of CA1 area from HIBD group showed only slightly decrease compare to the sham + DHA group (Fig. 2a, b) (RCNI^sham^ = 100 ± 1.09, RCNI^sham+DHA^ = 104.25 ± 1.18, RCNI^HIBD^ = 98.31 ± 1.01, RCNI^HIBD+DHA^ = 100.74 ± 1.18; n = 4, 3, 4, 5 for sham, sham + DHA, HIBD, and HIBD + DHA group, respectively; p_sham vs. HIBD_ > 0.05, p_sham+DHA vs. HIBD_ > 0.05, p_HIBD vs. HIBD+DHA_ > 0.05; Two-way ANOVA, Tukey’s multiple comparisons test).

In addition, the neuron number decrease in the deeper layers of cortex (layer IV-V) was also observed, and showed the similar HIBD-induce/DHA-rescue pattern with the one happened in hippocampal CA3 area (Fig. 2a, d) (RCNI^sham^ = 100 ± 2.19, RCNI^sham+DHA^ = 99.89 ± 2.85, RCNI^HIBD^ = 80.13 ± 3.54, RCNI^HIBD+DHA^ = 101.59 ± 2.72; n = 4, 3, 4, 5 for sham, sham + DHA, HIBD, and HIBD + DHA group, respectively; ***p_sham vs. HIBD_ = 0.0006, **p_sham+DHA vs. HIBD_ = 0.0012, ***p_HIBD vs. HIBD+DHA_ = 0.0002; Two-way ANOVA, Tukey’s multiple comparisons test).

### DHA treatment ameliorates the spatial learning and memory ability in HIBD rats

**Fig. 3:**
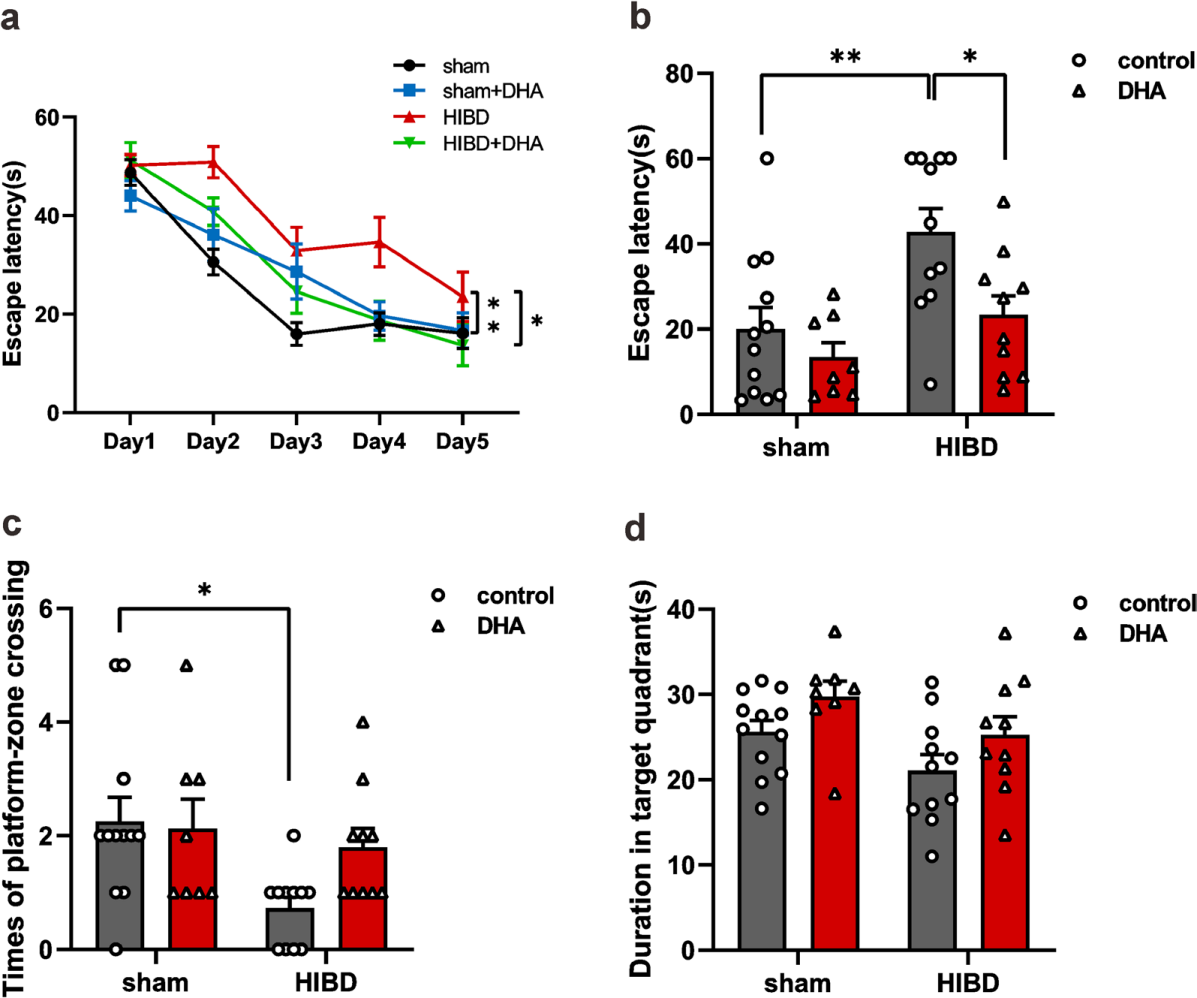
DHA treatment saves the spatial learning and memory ability of rats after HI. **a** Escape latency of rats for finding the hidden platform during training sessions (day1-day5) in the Morris water maze task. **b** Escape latency for finding the platform-zone in probe test. **c** Quantification of the times of the platform-zone crossing in probe test. **d** Quantification of the time rat spent in the target quadrant in probe test

Given that DHA prevents the HIBD induced neuron loss, we assumed that the spatial learning and memory of HIBD rats would also be protected by DHA. To prove our hypothesis, we tested the rats with Morris water maze. All rats were trained in the Morris water maze for 5 days. During the training, HIBD rats spent significantly longer time than rats from other groups to find the platform, although the average escape latency shortened down with practices as similar as other groups (Fig. 3a) (n = 12, 8, 11, 10 for sham, sham + DHA, HIBD, and HIBD + DHA group, respectively; **p_sham vs. HIBD_ = 0.001, *p_sham+DHA vs. HIBD_ = 0.017, *p_HIBD vs. HIBD+DHA_ = 0.019; Two-way ANOVA, Tukey’s multiple comparisons test). Next, memory for the platform location was probed by recording the time spent to reach the original platform-zone (escape latency), the times of platform-zone crossing, and the time spend in the original quadrant of the platform after the platform was removed. Results showed that the escape latency of HIBD rats was significantly longer than the control groups with 36% of them reached the 60 s cut-off time, while the data from rats with DHA treatment after HI was as similar as control groups (Fig. 3b) (sham = 20.00 ± 5.03 s, sham + DHA = 13.38 ± 3.37 s, HIBD = 39.53 ± 6.21 s, HIBD + DHA = 23.27 ± 4.58 s; **p_sham vs. HIBD_ = 0.0072, **p_sham+DHA vs. HIBD_ = 0.0015, *p_HIBD vs. HIBD+DHA_ = 0.0358; n = 12, 8, 11, 10 for sham, sham + DHA, HIBD, and HIBD + DHA group, respectively; Two-way ANOVA, Tukey’s multiple comparisons test). Interestingly, more than one third of rats in HIBD group couldn’t find the destination when we counted the times of platform-zone crossing (Fig. 3c) (sham = 2.25 ± 0.43, sham + DHA = 2.13 ± 0.52, HIBD = 0.73 ± 0.19, HIBD + DHA = 1.8 ± 0.33; *p_sham vs. HIBD_ = 0.0210, p_sham+DHA vs. HIBD_ > 0.05, p_HIBD vs. HIBD+DHA_ > 0.05; n = 12, 8, 11, 10 for sham, sham + DHA, HIBD, and HIBD + DHA group, respectively; Two-way ANOVA, Tukey’s multiple comparisons test). However, there was no significant difference in time spent in the target quadrant within 60 s (Fig. 3d) (sham = 25.58 ± 1.38 s, sham + DHA = 29.70 ± 1.88 s, HIBD = 21.05 ± 1.88 s, HIBD + DHA = 25.26 ± 2.15 s; p_sham vs. HIBD_ > 0.05, p_sham+DHA vs. HIBD_ > 0.05, p_HIBD vs. HIBD+DHA_ > 0.05; n = 12, 8, 11, 10 for sham, sham + DHA, HIBD, and HIBD + DHA group, respectively; Two-way ANOVA, Tukey’s multiple comparisons test). Together, these data indicate that HIBD rats have difficulties to remember the position of the platform, but rats with DHA treatment after HI had better performance in these tests.

### DHA treatment rescues myodynamia and locomotor functions in HIBD rats

**Fig. 4:**
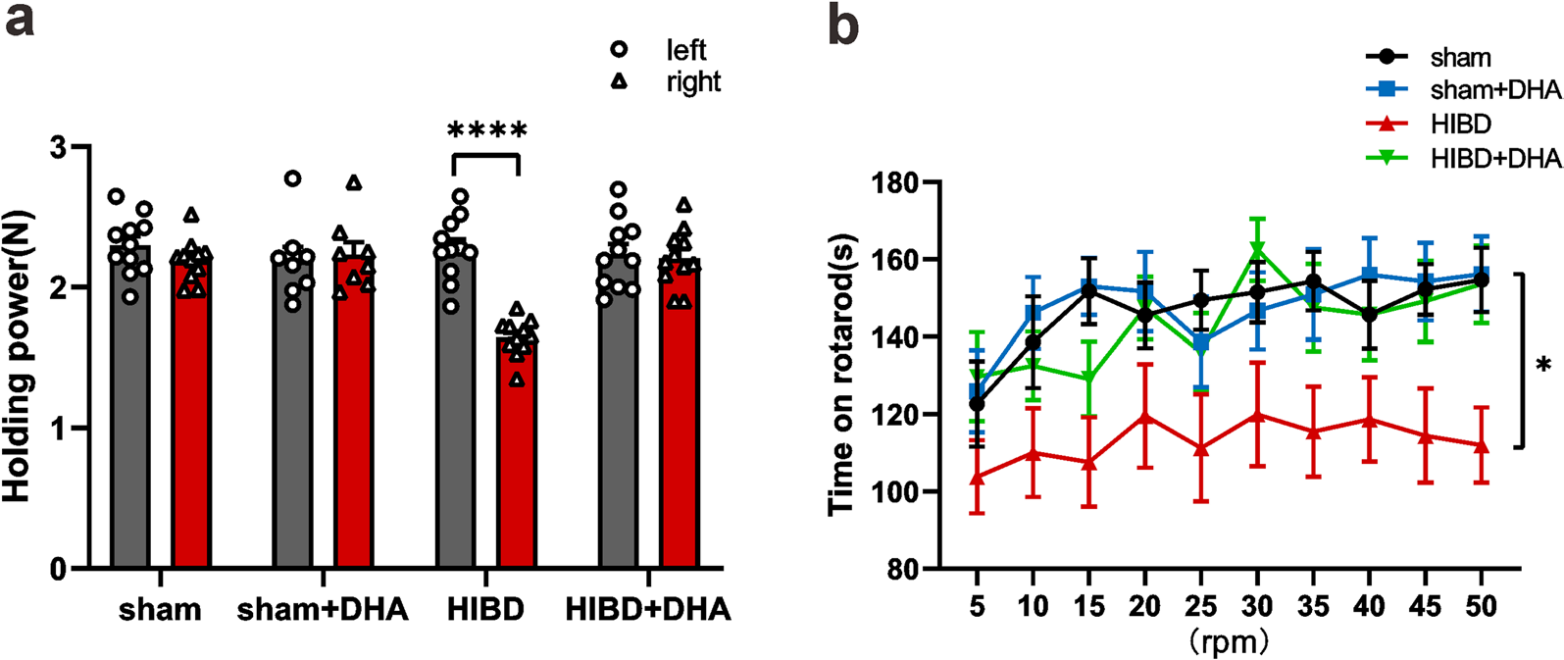
DHA treatment ameliorates the front limb holding power motor function of rats after HI. **a** Quantification comparisons of bilateral forelimbs’ strength of rats in each group. **b** Quantification of the time spent by rats on rotarods (x axis shows the rotary speed of test)

Patients with HIBD are also reported to have impaired motor ability, as do many mouse models [35–37]. To evaluate the myodynamia and locomotor condition of rats, grasping test and rotarod performance test were proceeded. In grasping test, the muscle strength of rats’ right forelimbs was significantly lower than the left ones in HIBD group (Fig. 4a) (holding power: left = 2.27 ± 0.07 N, right = 1.64 ± 0.04 N; n = 11, ****p < 0.0001; Two-way ANOVA, Tukey’s multiple comparisons test), while rats of other three groups, especially the HIBD + DHA group, did not have this effect (Fig. 4a) (n = 11, 8, 10 for sham, sham + DHA and HIBD + DHA groups, respectively; Two-way ANOVA, Tukey’s multiple comparisons test). Similarly, in the rotarod test, HIBD rats spent significantly less time on the rotating rod compared to the rats in other three groups (Fig. 4b) (n = 12, 8, 11, 10 for sham, sham + DHA, HIBD and HIBD + DHA groups, respectively; *p_sham vs. HIBD_ = 0.0171; *p_sham+DHA vs. HIBD_ = 0.0287; *p_HIBD vs. HIBD+DHA_ = 0.0496; Two-way ANOVA, Tukey’s multiple comparisons test). These results suggest that DHA treatment was helpful to protect the rats’ coordination and motor ability from HIBD.

### DHA treatment reduces oxidative stress levels in the brain of HIBD rats

**Fig. 5:**
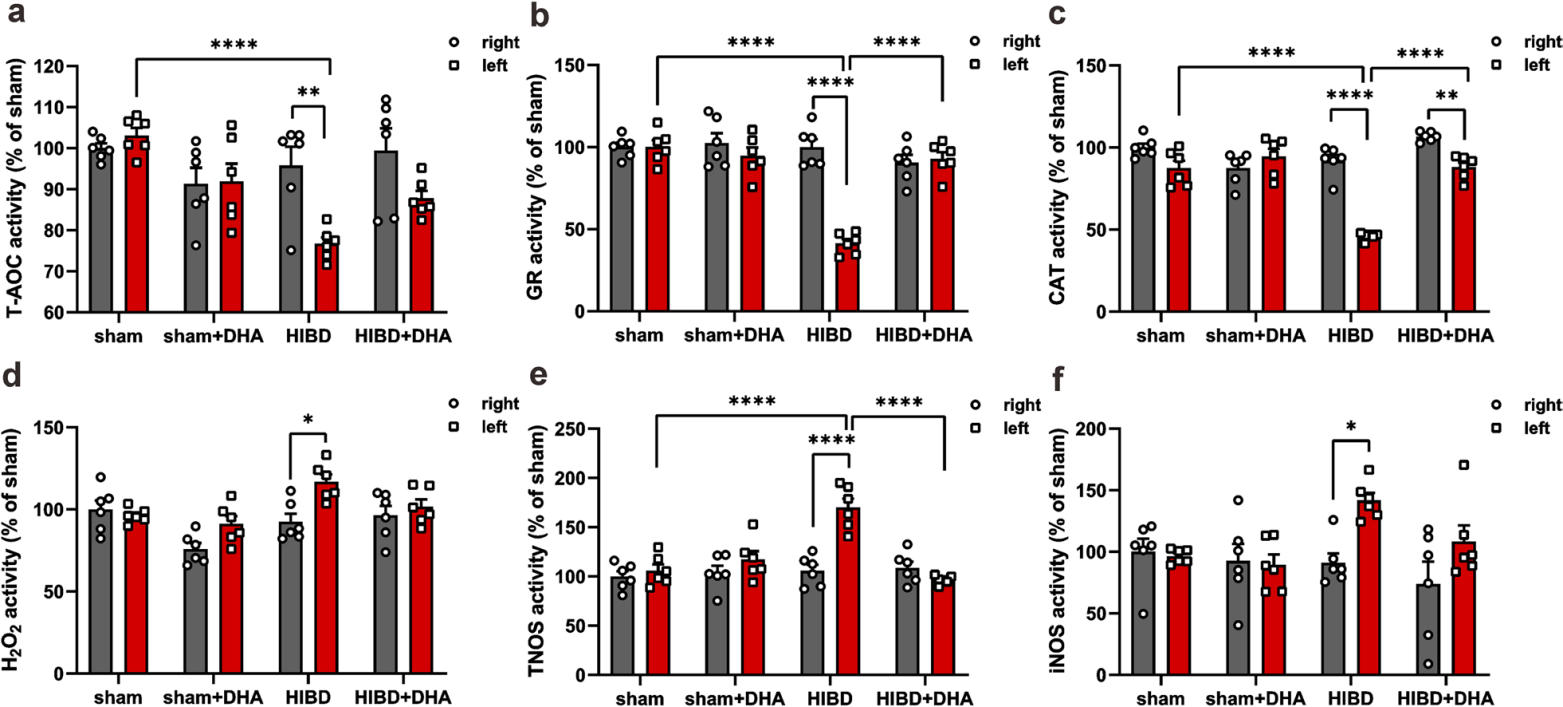
DHA treatment increases the antioxidants level and decrease the pro-oxidants level of HIBD rat brains. **a**–**c** Quantification of antioxidants T-AOC, GR and CAT in bilateral cerebral hemispheres. **d**–**f** Quantification of pro-oxidants H_2_O_2_, TNOS and iNOS in bilateral brain tissues

To explore the underlying protective mechanism(s) of DHA to HIBD, we measured the oxidation and antioxidation ability of bilateral rat brains after 2 days (P9) of HI. Our results showed that the antioxidants (T-AOC, GR, CAT) from the left hemispheres of HIBD rats (HIBD-L) were significantly decreased (Fig. 5a–c) with markedly increased pro-oxidative substances (H_2_O_2_, TNOS, iNOS) (Fig. 5d–f) compared to the right ones (HIBD-R) or the ipsilateral hemispheres of sham group (sham-L), indicating the imbalance of oxidative reaction after HI. As expected, the trend of DHA group reversed, compared to the HIBD group, to the pattern as similar as sham and sham + DHA groups (Fig. 5a–f). All data were normalized with the average value of right brains in sham group (sham-R) (For T-AOC: sham-R = 100 ± 1.18%, sham-L = 103.10 ± 1.81%, sham + DHA-R = 91.31 ± 3.90%, sham + DHA-L = 91.87 ± 4.36%, HIBD-R = 95.79 ± 4.58%, HIBD-L = 76.72 ± 1.58%, HIBD + DHA-R = 99.37 ± 5.46%, HIBD + DHA-L = 87.76 ± 1.88%; ****p_sham-L vs. HIBD-L_ < 0.0001, **p_HIBD-R vs. HIBD-L_ = 0.0078, p_HIBD+DHA-L vs. HIBD-L_ > 0.05, p_HIBD+DHA-R vs. HIBD+DHA-L_ > 0.05; For GR: sham-R = 100 ± 2.67%, sham-L = 100.14 ± 4.01%, sham + DHA-R = 102.39 ± 6.01%, sham + DHA-L = 94.66 ± 5.03%, HIBD-R = 99.89 ± 4.88%, HIBD-L = 41.26 ± 2.67%, HIBD + DHA-R = 90.49 ± 4.80%, HIBD + DHA-L = 92.86 ± 4.08%, ****p_sham-L vs. HIBD-L_ < 0.0001, ****p_HIBD-R vs. HIBD-L_ < 0.0001, ****p_HIBD+DHA-L vs. HIBD-L_ < 0.0001, p_HIBD+DHA-R vs. HIBD+DHA-L_ > 0.05; For CAT: sham-R = 100 ± 2.45%, sham-L = 87.49 ± 4.41%, sham + DHA-R = 87.55 ± 3.92%, sham + DHA-L = 94.59 ± 4.64%, HIBD-R = 91.86 ± 3.71%, HIBD-L = 45.92 ± 0.95%, HIBD + DHA-R = 106.72 ± 1.16%, HIBD + DHA-L = 88.10 ± 2.87%; ****p_sham-L vs. HIBD-L_ < 0.0001, ****p_HIBD-R vs. HIBD-L_ < 0.0001, ****p_HIBD+DHA-L vs. HIBD-L_ < 0.0001, **p_HIBD+DHA-R vs. HIBD+DHA-L_ = 0.0059; For H_2_O_2_: sham-R = 100 ± 5.52%, sham-L = 96.25 ± 1.89%, sham + DHA-R = 75.82 ± 3.71%, sham + DHA-L = 91.20 ± 4.80%, HIBD-R = 92.46 ± 4.89%, HIBD-L = 116.82 ± 4.54%, HIBD + DHA-R = 96.39 ± 5.83%, HIBD + DHA-L = 101.66 ± 4.49%; p_sham-L vs. HIBD-L_ > 0.05, *p_HIBD-R vs. HIBD-L_ = 0.0122, p_HIBD+DHA-L vs. HIBD-L_ > 0.05, p_HIBD+DHA-R vs. HIBD+DHA-L_ > 0.05; For TNOS: sham-R = 100 ± 5.38%, sham-L = 105.84 ± 6.28%, sham + DHA-R = 104 ± 6.97%, sham + DHA-L = 117.39 ± 8.31%, HIBD-R = 105.73 ± 6.23%, HIBD-L = 170.26 ± 8.87%, HIBD + DHA-R = 108.71 ± 6.46%, HIBD + DHA-L = 96.59 ± 1.75%; ****p_sham-L vs. HIBD-L_ < 0.0001, ****p_HIBD-R vs. HIBD-L_ < 0.0001, ****p_HIBD+DHA-L vs. HIBD-L_ < 0.0001, p_HIBD+DHA-R vs. HIBD+DHA-L_ > 0.05; For iNOS: sham-R = 100 ± 10.58%, sham-L = 96.36 ± 2.33%, sham + DHA-R = 92.64 ± 13.83%, sham + DHA-L = 89.38 ± 8.38%, HIBD-R = 91.11 ± 7.44%, HIBD-L = 141.73 ± 6.13%, HIBD + DHA-R = 73.85 ± 18.16%, HIBD + DHA-L = 108.23 ± 13.15%; p_sham-L vs. HIBD-L_ > 0.05, *p_HIBD-R vs. HIBD-L_ = 0.0446, p_HIBD+DHA-L vs. HIBD-L_ > 0.05, p_HIBD+DHA-R vs. HIBD+DHA-L_ > 0.05; For all groups: n = 6; Two-way ANOVA, Tukey’s multiple comparisons test).

## Discussion

In this study, we demonstrated the protective effect of DHA for saving the rat brains from HIBD, showed the decreased infarcted area and neuron loss after HI with DHA treatment. Furthermore, the spatial learning and memory, limb strength and motor coordination activity also been saved by DHA treatment after HI. Biochemistry tests showed one of the underlying mechanisms would be the increasing level of antioxidants and decreasing level of pro-oxidants by DHA treatment.

Neuronal electric activity is positively related to glucose metabolism rate, cerebral blood flow and vascular density both in mature and immature brains of mammals [38]. The aerobic metabolism of glucose is blocked and adenosine triphosphate (ATP) synthesis is reduced in hypoxic-ischemic encephalopathy. As results of energy deficiency, glutamate increasing in synaptic gap, excitotoxicity and huge amount of calcium ion influx lead to oxidative stress [39–41]. Furthermore, mitochondria, as the most important organelle to generate ROS by oxidative respiratory chain after HI [42, 43], dysfunction with the excessive calcium influx and further aggravate energy deficiency [44, 45]. Additionally, microglia cells are activated when HI occurs, generating oxygen free radicals, promoting the release of inflammatory mediators, take IL-1β as an example, which could interact with activated microglia and lead to immune cascade reaction [46, 47]. All above induce irreversible damages to the brain, prove that oxidative stress plays an important role during the development of HIBD.

As to necrosis, it is a type of uncontrolled cell death usually triggered by severe and/or sustained hazards as acute trauma, energy failure/ischemia, excitotoxicity and so on [48, 49]. Morphologically, necrosis usually results to cytoplasmic swelling (oncosis), cytoplasmic organelles swelling, membrane blebbing and disruption, nuclear disruption (karyolysis), leading the cells to disruption [50, 51]. As a result, cell lysate would be released into the extracellular compartment, leading to the damage of neighboring cells [52]. In our study, hypoxia and ischemia was considered as the major cause of the necrosis as the HIBD rats were used. The DHA injection right after HIBD modeling surgery prevented or significantly slowed the expanding damage caused by HI, and thus inhibited HI-induced necrosis. These evidences reversibly confirmed the protective effect of DHA in HIBD.

In this study, DHA treatment (50 mg/kg) significantly improved the behavioral performance of rats after HI, saved the loss of neurons and partially restored the balance between pro-oxidation and anti-oxidation. However, long-term blood exposure with high concentration of DHA is reported to have neurotoxicity [53, 54]. So, the concentration gradient of DHA (0, 5, 20, 50 mg/kg) was tested by intraperitoneal injection due to higher bioavailability and faster absorption rate. As a result, behavioral tests showed that the therapeutic effect of 50 mg/kg DHA treatment group was the best compare to other groups (data not shown). Thus, we picked 50 mg/kg DHA treatment as the optimum dose for rats in our study, but it needs to be adjusted according to the object in the research in future.

Furthermore, the optimum time window and concentration combination of DHA treatment need to be further studied with pharmacokinetics and the time when the imbalance between oxidation and antioxidation happens after HI. It only takes few minutes to cause permanent brain damage for global HI. But in our study, the time for irreversible impairment would be longer because of the compensatory effect from the non-ligated side.

Additionally, the number of NeuN^+^ cells in hippocampal CA3 area of HIBD group was significantly less than that of other groups, while no difference in CA1 area was observed in this study. This phenotype in hippocampus is inconsistent with our previous reported HIBD models which had neuron loss in different hippocampal areas at the same age [27]. We speculate this as the systematically difference of the surgery when modeling the HIBD rats by different operator as it was consistent in the rats modeled by the same operator. However, this do not affect the conclusion that the DHA treatment (50 mg/kg/day as described in Fig. 1a) could significantly prevent the HI induced neuron loos.

The effect of current common treatment for HIBD, including supportive therapy, controlling convulsion, reducing intracranial pressure, mild hypothermia therapy, rehabilitation training, and cell therapy, is not sufficient enough [55–57]. In this study, we provide DHA treatment as a potential treatment for HIBD patients.

## Conclusion

In summary, our data proved that DHA treatment suppressed HI-induced oxidative stress and rescued neuron loss so as to improve motor, learning and memory abilities in rats with HIBD, indicating that DHA is promising to be one of the therapeutic drugs for HIBD.

## Data Availability

Data sharing not applicable to this article as no datasets were generated or analyzed during the current study.

## Abbreviations

DHA: Dihydroartemisinin
HIBD: Hypoxic-ischemic brain damage
HIE: Hypoxic-ischemic encephalopathy
HI: Hypoxia ischemia
T-AOC: Total antioxidant capacity
GSH: Glutathione
GR: Gluathione reductase
CAT: Catalase
H_2_O_2_: Hydrogen peroxide
TNOS: Total nitric oxide synthase
iNOS: Inducible nitric oxide synthase
ROS: Reactive oxygen species
SOD: Superoxide dismutase
AMPK: Adenosine 5ʹ-monophosphate-activated protein kinase
Nrf2: Nuclear factor erythroid-2-related factor 2
NF-κB: Nuclear factor kappa B
BDNF: Brain-derived neurotrophic factor
SD: Sprague-Dawley
RT: Room temperature
O_2_: Oxygen
N_2_: Nitrogen
TTC: 2,3,5-Triphenyltetrazolium chloride
IF: Immunofluorescence
DMSO: Dimethyl sulfoxide
PBS: Phosphate-buffered saline
℃: Degree centigrade
h: Hour
min: Minute
µm: Micrometer
mg: Milligram
kg: Kilogram
NE: Northeast
SE: Southeast
SW: Southwest
NW: Northwest
RCNI: Relative cell number index
ROI: Region of interest
ATP: Adenosine triphosphate
